# Chimeric RHDV Virus-Like Particles Displaying Foot-and-Mouth Disease Virus Epitopes Elicit Neutralizing Antibodies and Confer Partial Protection in Pigs

**DOI:** 10.3390/vaccines9050470

**Published:** 2021-05-07

**Authors:** Giselle Rangel, Juan Bárcena, Noelia Moreno, Carlos P. Mata, José R. Castón, Alí Alejo, Esther Blanco

**Affiliations:** 1 Centro de Investigación en Sanidad Animal (CISA, CSIC-INIA), Valdeolmos, 28130 Madrid, Spain; grangel@indicasat.org.pa (G.R.); barcena@inia.es (J.B.); noeliamorm@gmail.com (N.M.); alejo@inia.es (A.A.); 2 Department of Structure of Macromolecules, Centro Nacional de Biotecnología/CSIC, Cantoblanco, 28049 Madrid, Spain; c.perezmata@leeds.ac.uk (C.P.M.); jrcaston@cnb.csic.es (J.R.C.)

**Keywords:** virus-like particles (VLPs), chimeric VLPs, nanoparticles, vaccine platform, RHDV, FMDV, B-cell epitope, T-cell epitope, immune response, pig

## Abstract

Currently there is a clear trend towards the establishment of virus-like particles (VLPs) as a powerful tool for vaccine development. VLPs are tunable nanoparticles that can be engineered to be used as platforms for multimeric display of foreign antigens. We have previously reported that VLPs derived from rabbit hemorrhagic disease virus (RHDV) constitute an excellent vaccine vector, capable of inducing specific protective immune responses against inserted heterologous T-cytotoxic and B-cell epitopes. Here, we evaluate the ability of chimeric RHDV VLPs to elicit immune response and protection against Foot-and-Mouth disease virus (FMDV), one of the most devastating livestock diseases. For this purpose, we generated a set of chimeric VLPs containing two FMDV-derived epitopes: a neutralizing B-cell epitope (VP1 (140–158)) and a T-cell epitope [3A (21–35)]. The epitopes were inserted joined or individually at two different locations within the RHDV capsid protein. The immunogenicity and protection potential of the chimeric VLPs were analyzed in the mouse and pig models. Herein we show that the RHDV engineered VLPs displaying FMDV-derived epitopes elicit a robust neutralizing immune response in mice and pigs, affording partial clinical protection against an FMDV challenge in pigs.

## 1. Introduction

Virus-like particles (VLPs) are macromolecular assemblies with well-defined geometry that mimic the overall structure of the native virions, while lacking any viral genome [1,2]. These multimeric complexes form due to the intrinsic ability of viral capsid subunits to self-assemble into nanoparticles [3,4]. They are composed of multiple copies of one or more viral proteins and are usually antigenically indistinguishable from infectious virus [5]. VLPs are appealing as vaccine candidates because their inherent properties (i.e., being virus-sized, displaying multimeric antigens, being highly organized and having a repetitive structure, being a self-adjuvanting immunogen, not being infectious) make them suitable for the induction of safe and efficient humoral and cellular immune responses [6,7,8,9]. VLP-based vaccines have already been licensed for human and veterinary diseases and numerous vaccine candidates are currently in late stages of evaluation [10,11,12]. Furthermore, the development of VLPs as platforms for foreign antigen display, by chemical or genetic engineering [13,14,15], has further broadened their potential applicability both as prophylactic and therapeutic vaccines [16,17,18]. This work focuses on the use of the rabbit hemorrhagic disease virus (RHDV) capsid as a platform for antigen display.

RHDV, a pathogen causing a highly infectious and fatal disease of the European rabbit, is the prototype species of the *Lagovirus* genus within the *Caliciviridae* family [19]. It is a non-enveloped, icosahedral, single-stranded positive-sense RNA virus. The virus capsid (~40 nm diameter) comprises 180 copies, organized as 90 dimers, of the single capsid subunit, VP60 (also termed VP1), arranged on a T = 3 icosahedral lattice [20]. Due to the lack of an effective cell culture system for the propagation of RHDV, much of our understanding of this virus relies on studies performed with recombinant VLPs that are morphologically and antigenically identical to infectious RHDV virions [21]. RHDV VLPs have been shown to induce full protection of rabbits against a lethal challenge [21].

**Figure 1:**
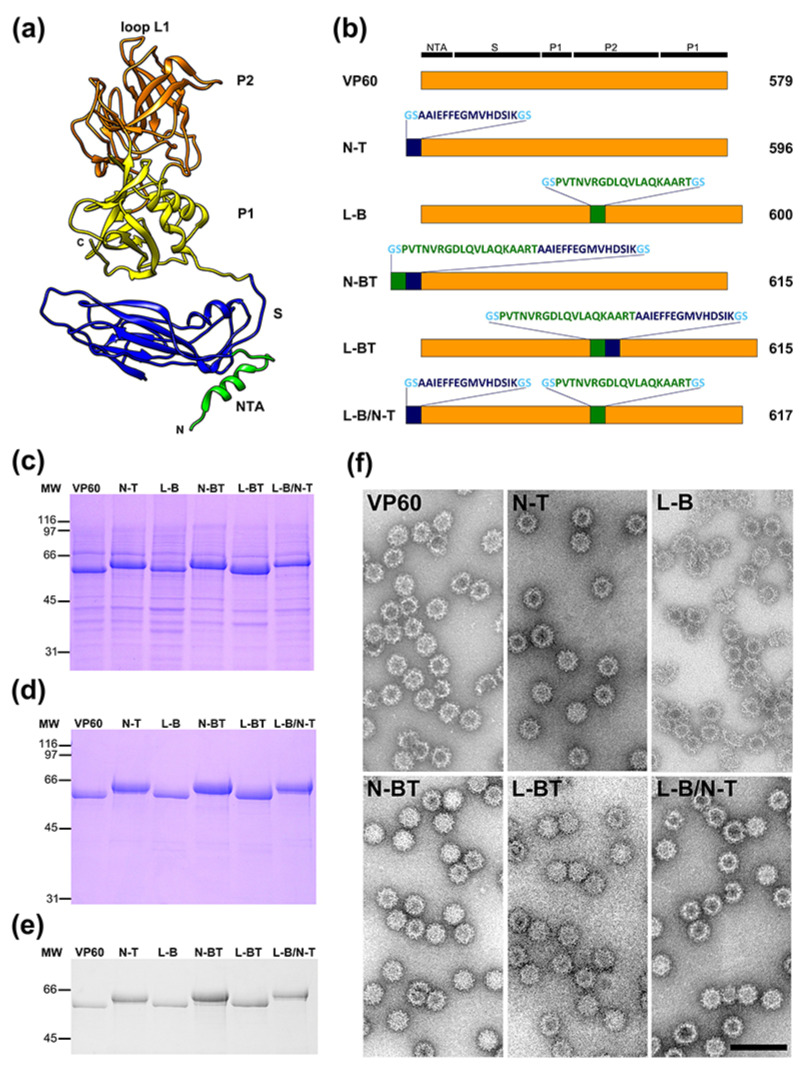
Expression and characterization of VP60 insertion mutants harboring FMDV B- and T-cell epitopes. (**a**) Ribbon representation of the VP60 protein structure (Protein Data Bank (PDB) accession number 3J1P). The NTA, S domain, P1 and P2 subdomains, and loop L1 are indicated. (**b**) Schematic representation showing names (left) and protein lengths in amino acids (right). The amino acid sequences depicted (B-cell epitope, T-cell epitope and B and T-cell epitope in tandem) were inserted at the indicated positions in each VP60 insertion mutant. RHDV capsid protein (VP60) is also shown. (**c**) H5 cells were infected with each recombinant baculovirus and infected-cell lysates were analyzed by SDS-PAGE as indicated. (**d**) Infected H5 cell cultures were subjected to VLP purification procedures and the resulting samples were characterized by SDS-PAGE. (**e**) Western blotting performed with the purified samples using a rabbit hyperimmune serum against RHDV to detect VP60 protein. The position of molecular weight markers (MW; ×10^3^ Da) are shown on the left. (**f**) Analyses of VP60-related VLPs by electron microscopy. Negatively stained purified particles corresponding to RHDV VP60 and the indicated VP60 chimeric mutants were analyzed by electron microscopy. Scale bar = 100 nm.

The VP60 protein has three domains [22] (Figure 1a): an internally located N-terminal arm (NTA), a shell domain (S) forming a continuous scaffold, and a flexible protruding domain (P) at the capsid surface, containing determinants for virus–host receptor interactions and antigenic diversity [22,23,24]. The P domain can be further divided into P1 and P2 subdomains, with the P2 subdomain located at the outermost surface-exposed region of the viral capsid. Our previous structural analysis of the RHDV capsid showed that the VP60 protein can accommodate the insertion of foreign amino acid sequences (i.e., heterologous epitopes) at different insertion sites without disrupting its ability to assemble into VLPs [13,25,26,27], raising the possibility of using RHDV VLPs as foreign epitope carriers for vaccine development.

Foot-and-mouth disease (FMD) is a highly infectious disease of cloven-hoofed animals, being one of the most important livestock diseases in terms of economic impact [28]. Current chemically inactivated whole-virus vaccines have been proven effective but present relevant limitations, including biosafety concerns [29]. Consequently, there is a strong need for the development of alternative, efficient and safe FMD vaccines. In collaboration with other groups, we have previously worked on peptide-based approaches as a new strategy for FMD vaccine development. We used two immunogenic epitopes from an FMDV isolate of serotype O: the main B-cell antigenic site in FMDV located at the GH loop of FMDV capsid protein VP1 (residues aa 140–158) [30,31], and a sequence spanning residues 21–35 of the FMDV nonstructural protein 3A, encompassing a T-cell epitope which is immunodominant in domestic pigs [32]. We have reported that dendrimeric peptides, including two or four copies of the B-cell epitope linked to the T-cell epitope, elicit potent humoral and cellular specific responses, conferring solid protection to viral challenge in pigs [33,34,35], while juxtaposition of the same two epitopes in a linear peptide is significantly less efficient [36], confirming that multimerization is an important strategy to trigger an efficient immune response based on both FMDV epitopes.

With this in mind, in this report we evaluated the ability of RHDV VLPs as a vaccine vector for the induction of immune responses and protection against FMD. We generated a set of chimeric RHDV VLPs by inserting the two FMDV-derived epitopes either joined or individually at two different locations within the RHDV capsid protein (involving different levels of surface accessibility). The immunogenic potential of the different chimeric VLPs was analyzed in the mouse and pig models. The results obtained show that the RHDV engineered VLPs displaying FMDV-derived epitopes elicited a robust neutralizing immune response in mice and pigs, affording partial clinical protection against an FMDV challenge in pigs.

## 2. Materials and Methods

### 2.1. Virus, Cells and Peptides

Derivatives of Autographa californica nuclear polyhedrosis virus (AcMNPV) were used to obtain the recombinant baculoviruses expressing RHDV VP60-based constructs. Baculoviruses were propagated in Trichoplusia ni High five cells (H5) grown in monolayer cultures at 28 °C in TNM-FH medium (Sigma-Aldrich), supplemented with 5% fetal calf serum (Gibco, Waltham, MA, USA).

A virus stock derived from FMDV isolate O/UKG/11/01 (The Pirbright Institute, Surrey, UK) by three passages in swine kidney cells (IB-RS-2 cells), which maintained the consensus sequences at the capsid protein region, was used to perform virus neutralization assays and pig challenge experiments.

The B-cell (VP1 140–158; PVTNVRGDLQVLAQKAART) and T-cell (3A 21–35; AAIEFFEGMVHDSIK) epitopes derived from the FMDV O-UKG 11/01 isolate [37] were prepared as synthetic peptides by solid phase synthesis protocols and purified by HPLC (D. Andreu, Pompeu Fabra University, Barcelona, Spain).

### 2.2. Generation of Recombinant Baculovirus Transfer Vectors

Plasmid pHAPh306GS [13], containing the coding sequences of proteins VP60 and VP2, and the 3′ untranslated region of RHDV (strain AST/89, GenBank accession code Z49271), was used as a starting point to generate vectors corresponding to VP60 constructs harboring foreign amino acid sequences (FMDV-derived B and T-cell epitopes) at different locations, by Q5 Site-Directed mutagenesis (NEB), according to the manufacturer’s recommendations. The following vectors were generated: plasmid pAH104 (N-T construct), with the coding sequence of peptide GSAAIEFFEGMVHDSIKGS, containing the FMDV T-cell epitope [37], inserted at the N-terminal end; plasmid pAH105 (L-B construct), with the coding sequence of peptide GSPVTNVRGDLQVLAQKAARTGS, encompassing the FMDV B-cell epitope [37], inserted at the exposed loop L1 within P2 subdomain (Figure 1a), between amino acid positions 306 and 307 of VP60 protein; plasmids pAH106 and pAH107 (N-BT and L-BT constructs) with the coding sequence of peptide GSPVTNVRGDLQVLAQKAARTAAIEFFEGMVHDSIKGS, containing both B and T-cell epitopes, inserted at the N-terminal end or at loop L1, respectively; and plasmid pAH108 (L-B/N-T construct), with the coding sequence of peptide GSAAIEFFEGMVHDSIKGS (T-cell epitope) at the N-terminal end, and the coding sequence of peptide GSPVTNVRGDLQVLAQKAARTGS (B-cell epitope) at loop L1. The sequence of the complete modified VP60 coding sequences in all final plasmids was checked for absence of unwanted mutations.

### 2.3. Generation of Recombinant Baculoviruses

Recombinant baculoviruses were obtained by cotransfection of the corresponding baculovirus transfer vectors with the flashBACULTRA baculoviral genome (Oxford expression technologies, Oxford, UK), following the manufacturer’s instructions.

### 2.4. Expression and Purification of Recombinant Chimeric RHDV VLPs

H5 cell monolayers were infected with recombinant baculoviruses at a multiplicity of infection (MOI) of 10. At 4 days post-infection, the infected cells were collected by low speed centrifugation, and washed three times with 0.2 M phosphate-buffered saline for VLPs (PBS-V, 0.2 M sodium phosphate, 0.1 M NaCl, pH 6.0) containing protease inhibitors (Complete, Roche, Penzberg, Germany). Next, the pelleted cells were resuspended in distilled water, subjected to mild sonication and treated with DNAse I (Roche Applied Science, Penzberg, Germany) for 1 h at room temperature. Subsequently, samples were adjusted to 2% Sarkosyl (sodium N-lauroylsarcosine, Sigma, Waltham, MA, USA), 5 mM EDTA in PBS-V, and incubated overnight at 4 °C. Cell lysates were then clarified by low-speed centrifugation and the supernatant was centrifuged at 139,000× *g* for 2 h using a Beckman SW28 rotor. The pelleted material was subjected to centrifugation through a 20% (wt/vol) sucrose cushion in PBS-V at 148,000× *g* for 2.5 h using a Beckman SW55 rotor. Pellets were diluted in PBS-V and centrifuged at 139,000× *g* for 2 h using a Beckman SW28 rotor. Finally, samples were resuspended in PBS-V and stored at 4 °C. Protein concentrations of the VLP preparations were determined with a BCA protein assay kit, Pierce, Thermo Scientific (Waltham, MA, USA).

Proteins were resolved by SDS-polyacrylamide gel electrophoresis (PAGE) and were visualized by Coomassie brilliant blue staining.

### 2.5. Transmission Electron Microscopy

Samples (approximately 5 μL) were applied to glow discharged carbon-coated grids for 2 min and negatively stained with 2% (wt/vol) aqueous uranyl acetate. Micrographs were recorded with a Jeol 1200 EXII electron microscope operating at 100 kV at a nominal magnification of ×40,000.

### 2.6. Mice Immunization

Groups of five outbred Swiss mice (Swiss ICR-CD1, Harlan Laboratories, Indianapolis, IN, USA), 6 weeks old, female, were immunized through a subcutaneous route at days 0 and 22, with 100µg of the indicated VLP constructs emulsified in Montanide ISA 50V2 (Seppic, Paris, France) in a 1:1 (vol:vol) ratio and sacrificed at day 40. A negative control group was inoculated with PBS plus adjuvant. Blood samples were collected at day 0 (before priming), day 22 (before boost) and at day 40.

### 2.7. Immunization and Infections of Pigs

The immune response and protection conferred by a chimeric VLP was assessed using eight 9-week-old white cross-bred Landrace female pigs (Agropardal SA breed) which were free of antibodies to FMDV. Pigs were randomly assigned to a group of six animals (VLP immunized pigs) and a group of two animals (non-immunized pigs). Six pigs were immunized twice (days 0 and 19) by intramuscular injection with 2 mL of Montanide ISA 50V2 emulsion (Seppic, Paris, France) containing 1 mg (*n* = 3) or 0.1 mg (*n* = 3) of the chimeric VLP L-B/N-T. Animals were housed in separate units of the high-containment facility and challenged at day 66 with 5 × 10^3^ TCID_50_ of FMDV O/UKG/11/01, by inoculation at two sites of both main claws of the left-hindfoot pad (0.1ml/site). Animals were monitored daily for clinical signs and fever for 10 days and then euthanized at day 76.

A clinical score was calculated by the addition of points distributed as follows [36]: (i) an elevated body temperature of 40 °C (score of 1), >40.5 °C (score of 2), or > 41 °C (score 3); (ii) reduced appetite (1 point) or no food intake and food left over from the day before (2 points); (iii) lameness (1 point) or reluctance to stand (2 points); (iv) presence of heat and pain after palpation of the coronary bands (1 point) or not standing on the affected foot (2 points); (v) vesicles on the feet, dependent on the number of feet affected, with a maximum of 4 points; and (vi) visible mouth lesions on the tongue (1 point), gums or lips (1 point) or snout (1 point), with a maximum of 3 points. Animals were considered protected when lesions were only observed at the infection site [38]. Animals were considered partially protected when showing mild and delayed signs of disease (Clinical scores <8).

### 2.8. Detection of Specific Anti-RHDV-VLP Antibodies by ELISA

Antibodies against RHDV VP60 capsid protein were determined by enzyme-linked immunosorbent assay (ELISA) as previously described [13]. Polysorp 96-well ELISA plates (Nunc) were coated with purified RHDV VLPs (300 ng/well) diluted in 0.05 M carbonate–bicarbonate buffer (pH 9.6), and incubated overnight at 4 °C. Plates were blocked with PBS–5% skim milk (5% BLOTTO) for 1 h at 37 °C, washed three times with PBS–0.1% Tween 20 (PBS-T) and incubated with serial three-fold dilutions of each serum sample prepared in 3%BLOTTO, for 1 h at 37 °C. Two control samples (serial dilutions) were added to each plate: a monoclonal antibody specific against RHDV VP60 protein [24], and a serum sample known to lack VP60-specific antibodies. The test sera were analyzed in parallel in wells lacking antigen to determine background binding. After six washes with PBS-T, the plates were incubated at 37 °C for 45 min with HRP-conjugated goat anti-mouse IgG (Invitrogen, Carlsbad, CA, USA) for mice sera, or with Protein-A (Thermo Fisher, Waltham, MA, USA) for pig sera, both at 1:3000 dilution in 3% BLOTTO. Plates were extensively washed with PBS-T and a color reaction was developed with OPD (Sigma-Aldrich, Waltham, MA, USA). Reaction was stopped by addition of 3N H_2_SO_4_ and absorbance was read at 492 nm on a Fluostar Omega microplate reader. End point titers were reported as the reciprocal of the highest dilution that had an absorbance value greater than or equal to 0.2 O.D units above background (absorbance of wells lacking antigen, which were consistently in the range of 0.035–0.055, and never exceeded 0.100). 

### 2.9. Detection of Specific Anti-FMDV Antibodies by ELISA

Total anti-FMDV antibodies were determined by ELISA as described [35]. Assays were performed in 96-well high binding plates (Corning, New York, NY, USA), coated with 1 μg/well of synthetic peptide B overnight at 4 °C. Duplicate three-fold dilution series of each serum sample (mice or pigs) were prepared starting at 1:100. Pre-immune sera from VLP-immunized mice and pigs, as well as sera from non-immunized animals, were used as negative controls. A color reaction was developed with TMB (Invitrogen) and the reaction was stopped by addition of 1.8N H_2_SO_4_. End point antibody titers were expressed as the reciprocal of the last dilution giving the absorbance recorded in the control wells (serum at day 0) plus 2× standard deviation (SD).

### 2.10. Virus Neutralization Test (VNT)

Virus-neutralizing activity was determined in sera by a standard microneutralization test performed in 96-well plates, as described in the World Organisation for Animal Health (OIE) Manual of Diagnostic Tests and Vaccines for Terrestrial Animals [39]. Briefly, serial two-fold dilutions of each serum sample were incubated with 100 50% tissue culture infective doses (TCID_50_) of FMDV O/UKG/01 for 1 h at 37 °C. The remaining viral activity was determined in 96-well plates containing fresh monolayers of IB-RS-2 cells. End-point titers were calculated as the reciprocal of the final serum dilution that neutralized 100 TCID_50_ of FMDV O/UKG/01 in 50% of the wells [40].

### 2.11. IFN-γ Detection by ELISPOT

ELISPOT assays were performed essentially as described [37]. A mouse IFN-γ ELISPOT reagent kit was used according to manufacturer’s instructions (BD Biosciences, San Jose, CA, USA). After red blood cell lysis, 2.5 × 10^5^ splenocytes were distributed in triplicate wells of Immobilon-P hydrophobic PVDF 96-well plates (Millipore, S2EM004M99, Burlington, MA, USA), previously coated with an anti-mouse IFN-γ antibody, and then blocked with RPMI 1640 (Gibco), supplemented with glutamine, penicillin/streptomycin, and 10% inactivated FSB. Cells were stimulated with either FMDV T-peptide (20 µg/well), sucrose gradient-purified FMDV (O UKG 11/2001) (1 µg/well) or with 8 µg/well of purified VP60. Triplicate wells with 2.5 × 10^5^ cells without peptide were used to estimate nonspecific activation. As a positive control, triplicate wells with 2.5 × 10^5^ cells were stimulated with phytohemagglutinin (Sigma) at 80 µg/mL. After 48 h at 37 °C, 5% CO_2_ and 95% relative humidity, plates were washed and incubated with a biotinylated anti-mouse IFN-γ antibody followed by HRP-streptavidin. Antibody binding was visualized by the substrate 3-amino-9-ethylcarbazole. The frequency of peptide and virus-specific T cells present in the responding population was expressed as the mean number of spot-forming cells (SFC) counted in stimulated wells per 10^6^ splenocytes.

Swine IFN-γ ELISPOT was carried out as described [35]. Porcine PBMCs were obtained by density-gradient centrifugation with Histopaque 1077 (Sigma) and Leucosep tubes (Greiner Bio-One, Kremsmünster, Austria). Cells were collected and resuspended in complete RPMI 1640 and used fresh in the assays. Triplicate wells with 2.5 × 10^5^ cells were subjected to in vitro recall stimulation as described above for mice ELISPOT.

### 2.12. Lymphoproliferation Assay 

Swine lymphoproliferation assays were performed in sterile 96-well U-bottom plates, using a concentration of 3 × 10^5^ PBMCs/well. PBMCs were stimulated with the same conditions described in the ELISPOT-IFNγ assay. Concanavalin-A (Con-A) (2.5 µg/well) was used as a positive control and PBMCs incubated only with medium were used as a negative control. After 72 h at 37 °C, 5% CO_2_, another incubation period for 18 h with 0.1 μCi/100 μL of [3H]-Tymidine (Perkin Elmer, Life Science, Waltham, MA, USA) was performed. Incorporation of the radioactive marker into the cells was measured using a scintillation counter (Microbeta Counter Becton Dickinson). The mean value of the triplicates performed was calculated and the results are expressed as stimulation index (SI) (cpm stimulated cultures/cpm of the control without antigen).

### 2.13. Data and Statistical Analysis

Geometric mean titers (GMTs) of IgG in serum were determined for every group of mice and pigs. Differences in FMDV–antibody titers and numbers of IFNγ-producing cells among VLP-immunized groups were analyzed by one-way analysis of variance (ANOVA), followed by Tukey’s post hoc comparison tests. The statistical significance of differences in the clinical score values between VLP-immunized pigs and non-immunized pigs was calculated by Welch’s test. All *p* values are two-sided and *p* ≤ 0.05 were considered significant. In figures, *p* value criteria are assigned as * *p* < 0.05, ** *p* < 0.01, *** *p* < 0.001. Statistical analyses were performed using GraphPad Prism Software 6.0.

### 2.14. Ethics Statement

Animals were maintained at the animal facilities of the Centro de Investigación en Sanidad Animal (CISA CSIC-INIA) according to national and European Union guidelines for animal experimentation. The study received prior approval from the Ethical Committee for Animal Experimentation (CEEA2014/018) and Biosecurity Committee of INIA (CBS2014/015). All experimental procedures were conducted in accordance with protocols approved by the National Committee on Ethics and Animal Welfare (PROEX 218/14).

## 3. Results

### 3.1. Design and Characterization of RHDV-Based Chimeric VLPs Displaying FMDV B- and T-Cell Epitopes

We generated recombinant baculoviruses expressing different VP60 constructs (Figure 1b). Two foreign amino acid sequences were incorporated to the capsid protein: peptide GSAAIEFFEGMVHDSIKGS (19 aa), encompassing the FMDV T-cell epitope (residues 21–35 from the 3A protein) and peptide GSPVTNVRGDLQVLAQKAARTGS (23 aa) containing the FMDV B-cell epitope from serotype O (residues 140–158 from the VP1 capsid protein). The immunogenic epitopes inserted were flanked by the amino acids glycine and serine (GS) as a flexible linker intended to facilitate capsid assembly. On the basis of previous structural analyses [13,27], the foreign epitopes were inserted at two locations within the VP60 protein. Two chimeric mutants were generated by inserting either the T-cell epitope or both, the B and T cell epitopes in tandem, at the N-terminal end, between amino acid positions 2 and 3 of the VP60 protein sequence (N-T and N-BT constructs, respectively). According to the atomic structure of RHDV capsid [22], the N-terminus of VP60 protein faces the inner capsid surface. Two other chimeric mutants were produced by insertion of either the B-cell epitope or the B and T cell epitopes in tandem within loop L1, between amino acid positions 306 and 307 of VP60 protein (Figure 1a) (L-B and L-BT constructs, respectively). This insertion site lies at the tip of the P2 subdomain of VP60 protein, the most external region of the viral capsid [22]. An additional construct was generated bearing one copy of the T-cell epitope at the N-terminal end and one copy of the B-cell epitope at loop L1 (L-B/N-T).

Expression of the chimeric constructs in H5 insect cell cultures infected with the corresponding recombinant baculoviruses was analyzed by SDS-PAGE (Figure 1c). Extracts from cells infected with the recombinant baculovirus expressing VP60 parental protein exhibited a major protein band with an apparent molecular weight of ≈60 kDa. Expression levels of the VP60 insertion mutants were grossly similar to those of VP60 parental protein and the corresponding bands exhibited mobilities according to their expected molecular weights.

Subsequently, H5 cell cultures were infected with the recombinant baculoviruses and the infected cells were subjected to previously established VLP-purification procedures. The resulting samples were characterized by SDS-PAGE (Figure 1d) and Western blotting (Figure 1e), and were found to contain highly purified VP60-related proteins in all cases. Electron microscopic analysis additionally showed that all the chimeric constructs containing FMDV-derived epitopes assembled into VLPs of approximately 40 nm in diameter (Figure 1f) which were morphologically similar to the VLPs formed by the parental VP60 protein and to authentic virions, i.e., a capsid with a T = 3 icosahedral lattice.

### 3.2. Immune Responses Elicited by Chimeric VLPs Displaying FMDV B- and T-Cell Epitopes in Mice

#### 3.2.1. Chimeric VLPs Displaying FMDV Epitopes Elicit Specific Humoral Responses including Neutralizing Antibodies

**Figure 2:**
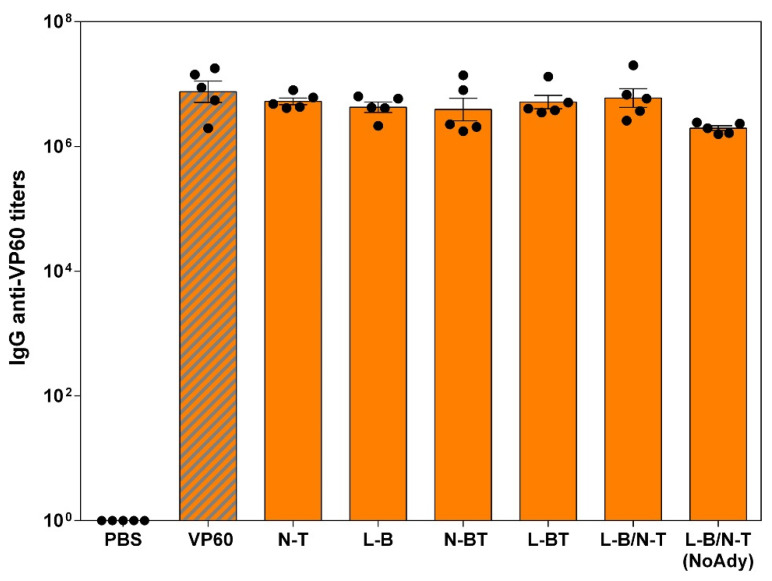
Serum IgG anti-VP60 antibodies elicited at day 40 measured by ELISA. The GMT was calculated for each group of mice. Error bars show the standard error of the mean. Each dot represents the value for an individual mouse. The group corresponding to mice inoculated with native RHDV VLPs (VP60) is shown as the hatched bar.

**Figure 3:**
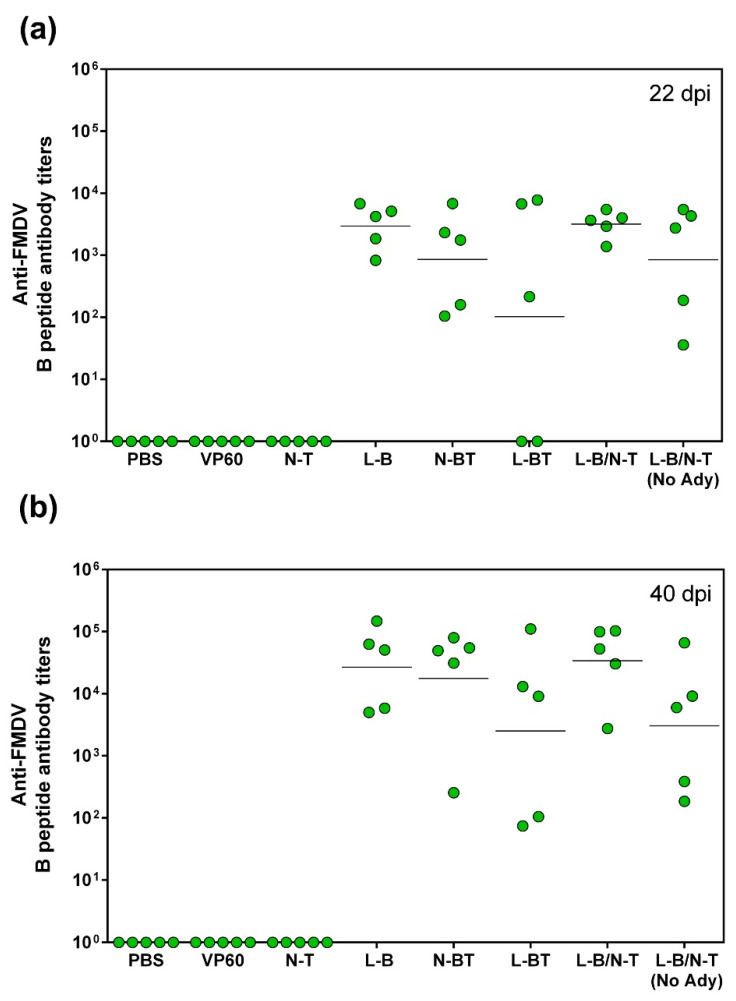
Serum IgG anti-FMDV antibodies measured by ELISA using a synthetic peptide encompassing a FMDV B-cell epitope sequence. Each symbol represents the value for an individual mouse (*n* = 5 per group). The GMT was calculated for each group (solid lines). (**a**) Anti-FMDV antibody titers elicited at day 22, after administration of a single dose of chimeric VLPs, (**b**) anti-FMDV antibody titers elicited at day 40 (18 days post-boost).

Groups of five Swiss ICR (CD-1) mice were immunized twice (at days 0 and 22) subcutaneously with 0.1 mg of each purified VLP (groups VP60, N-T, L-B, N-BT, L-BT, L-B/N-T) emulsified in Montanide ISA 50V2 adjuvant. An additional group was immunized with the chimeric VLP L-B/N-T without adjuvant. As a negative control, a group of mice was immunized with PBS with adjuvant. Blood samples were collected at days 0 (before priming), 22 (before boost) and 40 post-immunization and the sera obtained tested for antibodies against VP60 protein (RHDV VLPs) (Figure 2) or the FMDV-derived B-cell epitope (a synthetic peptide) (Figure 3a,b).

Serum IgG antibody titers were measured by ELISA, and geometric mean titers (GMTs) were calculated for each group of mice. All pre-immune serum samples, as well as sera from the control mice group receiving PBS with adjuvant were negative against both antigens tested.

As expected given the reported high immunogenicity of RHDV VLPs, all mice immunized with either RHDV or chimeric RHDV VLPs developed high titers of VP60-specific antibodies (GMTs ranging from 1.96 × 10^6^ to 7.54 × 10^6^, Figure 2) at day 40 post immunization (18 days post-boost).

Regarding the humoral response against FMDV, all chimeric RHDV VLPs bearing the B-cell epitope at any of the two insertion sites used elicited significant antibody titers against FMDV after a single dose (Figure 3a), which were boosted after administration of a second dose (Figure 3b), while sera from mice immunized with native RHDV VLPs or construct N-T, lacking the FMDV B-cell epitope, were negative.

Some differences among the specific antibody titers against FMDV were observed across groups. Mice immunized using chimeric VLPs with the B-cell epitope inserted individually (not in tandem) at loop L1 (constructs L-B and L-B/N-T), exhibited higher titers (GMT = 2966 and 3188, respectively), as well as lower dispersion within the group at day 22 (Figure 3a), than mice immunized with VLPs incorporating the B and T-cell epitope in tandem (N-BT and L-BT) either at the N-terminal end or at loop L1 of protein VP60 (GMT = 861 and 102, respectively). However, these differences were not statistically significant.

Interestingly, the chimeric VLP L-B/N-T administered without adjuvant elicited significant antibody titers against both VP60 and the FMDV B epitope, even after administration of a single dose (GMT = 849) (Figure 3a), confirming the inherently high immunogenicity of RHDV VLPs and their effectiveness as vaccination platforms [13,21,41].

The FMDV-specific antibody titers markedly increased following the second dose in all the groups of mice immunized with chimeric VLPs displaying the B-cell epitope (Figure 3b). The highest titers were induced by chimeric VLPs L-B and L-B/N-T (GMT = 26,798 and 34,025, respectively), although differences with the titers induced by the other chimeric VLPs were not statistically significant.

**Figure 4:**
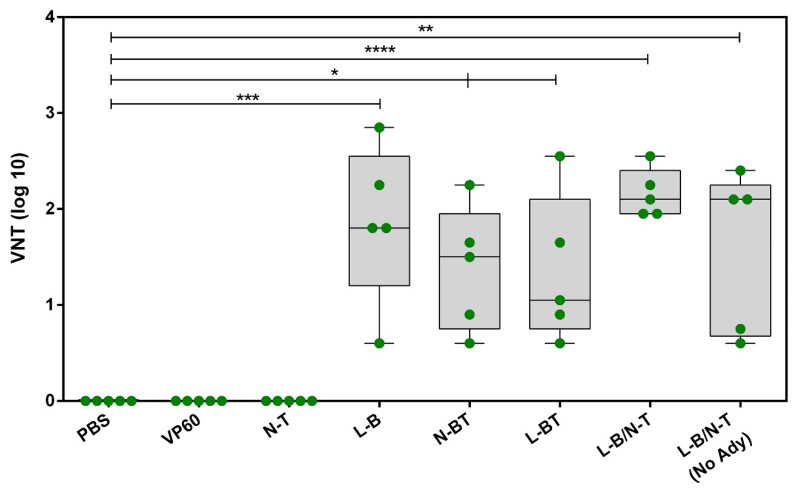
Box plot showing the distribution of FMDV-neutralizing antibody titers elicited by the chimeric VLPs. In this graphic, the box is defined by the interquartile range (IQR)—the 25th and 75th percentiles of the distribution; the horizontal line within the box represents the median (50th percentile) and the vertical lines extend to the extreme observations. Each symbol represents the value for an individual mouse (*n* = 5 per group). A value of 0 was assigned to all sera with VNT titers <0.6 (bellow detection limit). Statistically significant differences in FMDV VNT titers with respect to those corresponding to the VP60 group are shown as * *p* < 0.05, ** *p* < 0.01, *** *p* < 0.001, **** *p* < 0.0001. Identical statistically significant differences in FMDV VNT were found with respect to those corresponding to the N-T group (not shown in the graph for simplification of the figure).

Concerning the induction of FMDV neutralizing antibodies, serum samples collected at day 40 were analyzed by a virus neutralization test (VNT). As observed with the ELISA results, all the chimeric RHDV VLPs displaying the FMDV B-cell epitope elicited significant FMDV neutralizing activity, albeit to different extents (Figure 4). Negative control sera as well as sera from the groups of mice immunized with native VP60 RHDV VLPs and the chimeric construct N-T, failed to induce any detectable neutralizing activity. Mice immunized with chimeric VLP L-B/N-T exhibited the highest VNT titers, showing the lowest dispersion. Indeed, this was the only mice group in which the five animals exhibited VNT titers >2 log10 (Figure 4).

#### 3.2.2. T-Cell Responses Elicited by Chimeric VLPs Displaying FMDV Epitopes in Mice

**Figure 5:**
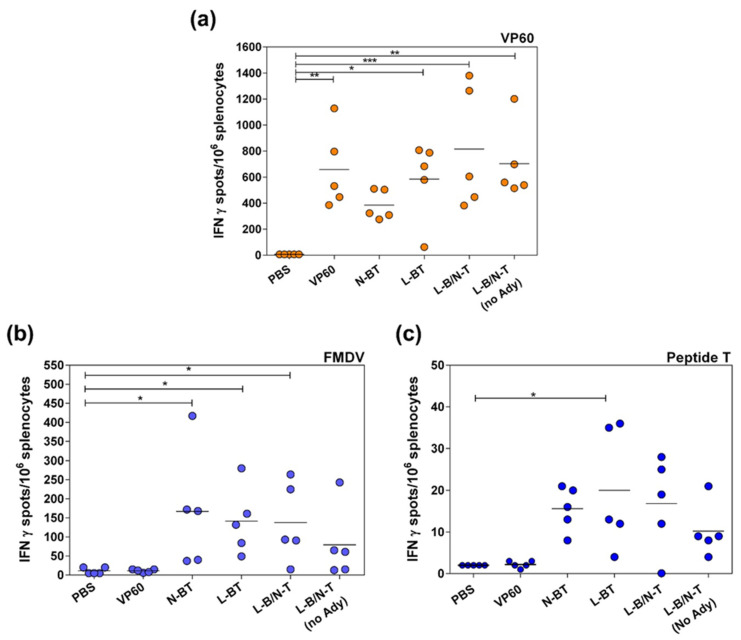
Specific T-cell responses in splenocytes from mice immunized with chimeric VLPs measured by an ex vivo IFN-γ ELISPOT. Splenocytes were stimulated in vitro with parental RHDV VP60 (**a**), with FMDV O-UKG (**b**) or with a synthetic peptide encompassing the FMDV T-cell epitope (**c**). Each symbol represents the mean value of triplicates of splenocytes for an individual mouse. Horizontal lines represent the mean frequencies of IFN-γ-releasing cells for each animal group. Significant differences of each group as compared to the reference PBS group are shown as * *p* < 0.05, ** *p* < 0.01, *** *p* < 0.001.

Specific T cell responses elicited by chimeric VLPs were determined at day 40 (18 days post-boost) by ELISPOT analyses of IFNγ-expressing splenocytes (Figure 5), after in vitro stimulation with: parental VP60 protein (Figure 5a), FMDV serotype O UKG/01 (Figure 5b) or FMDV T-cell peptide (Figure 5c). The assay was carried out with the groups of mice immunized with chimeric VLPs displaying both FMDV-derived epitopes (N-BT, L-BT and L-B/N-T). No specific IFNγ production was observed when cells from non-immunized mice (PBS group) were recalled in vitro with any of the stimuli used. IFNγ production was not detected either when splenocytes from mice immunized with parental RHDV VLPs (VP60 group) were stimulated in vitro with either FMDV virions or FMDV T-cell peptide. These results evidenced that the IFNγ production detected was antigen-specific.

A high frequency (>400 spots/10^6^ splenocytes) of RHDV VP60-specific IFNγ-secreting cells were observed in all groups of mice immunized with RHDV VLPs (parental or chimeric) (Figure 5a). Immunization of mice with RHDV VLPs (VP60 group) and chimeric constructs L-BT and L-B/N-T induced a statistically significant greater number of IFNγ-producing cells, in response to in vitro recall with RHDV VLPs, than the negative control (PBS group). The group of mice immunized with VLP L-B/N-T without adjuvant exhibited a similar frequency of VP60-specific IFNγ-secreting cells than the group immunized with the same VLP emulsified with adjuvant.

In contrast, the IFNγ responses elicited by chimeric VLPs after in vitro recall with FMDV antigens were detectable but weak. Mice immunized with chimeric VLPs N-BT, L-BT and L-B/N-T, developed mean frequencies of FMDV-specific IFNγ-secreting cells of 166.8 ± 154.6, 141.2 ± 88.76 and 137.6 ± 103.4, respectively, a statistically significant (*p* < 0.05) response with respect to the group of non-immunized mice (Figure 5b). Likewise, the chimeric VLPs elicited detectable but low quantities (<40 spots) of IFNγ in response to in vitro stimulation with the FMDV T-cell synthetic peptide (Figure 5c).

Overall, the results obtained demonstrate that the chimeric RHDV VLPs displaying FMDV-derived B and T-cell epitopes are able to induce a robust specific antibody response against FMDV in mice, including high titers of neutralizing antibodies. The construct L-B/N-T elicited the strongest and more consistent FMDV-neutralizing response. The chimeric VLPs were also able to induce detectable albeit modest specific T-cell responses to FMDV antigens.

### 3.3. Immune Response and Protection Conferred by Chimeric VLP L-B/N-T in Pigs

#### 3.3.1. VLP L-B/N-T Elicits Robust Humoral FMDV-Specific Response and VNT Titers in Pigs

On the basis of the results obtained from the mouse model, the chimeric VLP L-B/N-T was selected for further evaluation in swine, one of the most relevant FMDV natural hosts.

Six domestic pigs were vaccinated twice, at days 0 and 19, with chimeric VLP L-B/N-T. Three pigs were immunized using 1-mg doses of the chimeric VLP and the other three using 0.1-mg doses (emulsified with Montanide ISA 50V2). Subsequently, pigs were challenged, at day 66, with type O FMDV (isolate O/UKG/11/01). Two additional non-immunized pigs were included in the experiment as FMDV infection controls.

**Figure 6:**
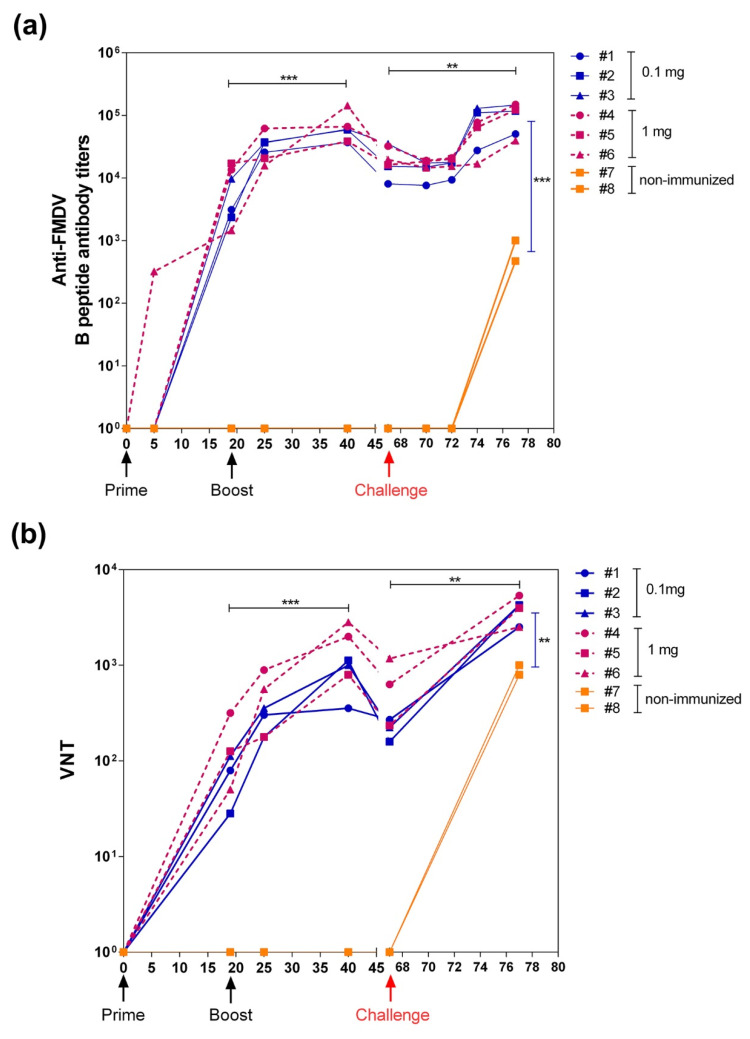
Kinetics of anti-FMDV antibody response elicited in pigs by the immunization with chimeric VLP and subsequent FMDV challenge. (**a**) Serum IgG anti-FMDV antibody titers measured by ELISA using a synthetic peptide encompassing the FMDV B-cell epitope sequence. (**b**) FMDV VNT titers. Sera below detection limit were arbitrarily assigned a titer of 1. Black arrows indicate the days of prime and boost immunization of the pigs with the chimeric VLP L-B/N-T. The red arrow indicates the day pigs were challenged with FMDV. Significant differences between antibody responses elicited in the six pigs before and after boost immunization and before and after challenge with FMDV are shown as ** *p* < 0.01, *** *p* < 0.001. Additionally, the significant differences between the antibody responses elicited 10 days post-challenge in the VLP-immunized pigs and the control non-immunized pigs are also shown.

Total FMDV-specific IgG antibodies were determined by ELISA using as antigen a synthetic peptide encompassing the B-cell epitope and serum samples collected at: 0, 5, 19, 25, 40, 67, 70, 72 and 77 days post-immunization. The results obtained (Figure 6a) indicated that both 0.1-mg and 1-mg doses of chimeric VLP elicited consistent and comparable IgG titers (GMT = 4153 and 6972, respectively) at day 19, after the administration of a single dose of chimeric VLP. Upon revaccination, all six immunized pigs exhibited a significant rise in serum IgG titers (GMT = 60,379) at day 40 (21 days post-boost). The titers further increased after FMDV challenge at day 77 (GMT = 92,591), while non-immunized control pigs exhibited significantly lower titers (GMT = 688) after challenge than those of the pigs that had been previously immunized with the chimeric VLP.

Since neutralizing antibodies are closely linked to protective immunity, the neutralizing response against FMDV over time was analyzed after prime/boost immunization and after FMDV challenge (Figure 6b). As observed with the ELISA results, similar VNT titers were elicited by immunization with 0.1 mg or 1 mg of chimeric VLP (GMT = 63 and 126, respectively) at day 19 post-immunization. After boost, FMDV VNT titers of the six immunized pigs significantly increased by day 40 (GMT = 1101) and were further increased after FMDV challenge by day 77 (GMT = 3673). In agreement with the ELISA results, the FMDV VNT titers of non-immunized pigs after challenge (GMT = 891) were significantly lower than those of the pigs previously immunized with the chimeric VLP.

#### 3.3.2. Specific Cellular Immune Responses Elicited by VLP L-B/N-T in Pigs

Specific T-cell responses elicited by the chimeric VLP were determined by ELISPOT analysis of IFN-γ producing PBMCs and by lymphoproliferation assay also using PBMCs.

ELISPOT assays of IFN-γ were performed after in vitro recall with synthetic peptides encompassing FMDV B or T cell epitopes, FMDV virions or parental RHDV VP60 protein at days 5, 19, 25 and 40 post-immunization.

**Figure 7:**
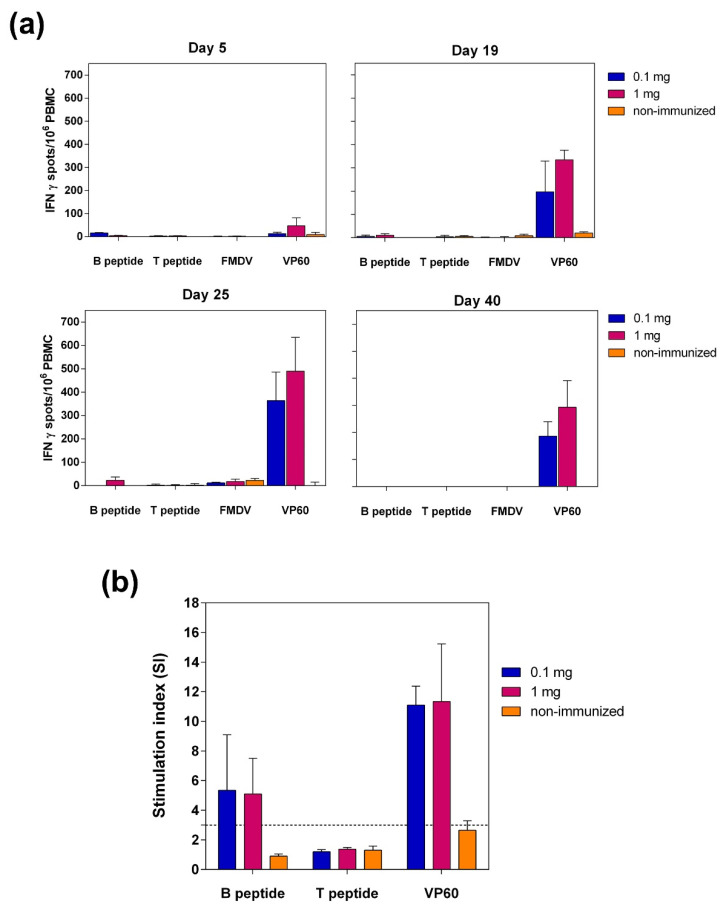
Specific T-cell responses in PBMCs from immunized pigs. (**a**) Frequency of IFNγ-secreting cells measured by ELISPOT at days 5, 19, 25 and 40 post-immunization, after in vitro recall with peptides (B or T), virus (FMDV) and RHDV VLPs (VP60). (**b**) Lymphoproliferation assay at day 25 post-immunization, after in vitro stimulation with peptides (B and T) and RHDV VLPs (VP60). The dashed line depicts the threshold above which the lymphoproliferative response is considered significant (>3).

Both 0.1- and 1-mg doses of chimeric VLP elicited high and comparable mean frequencies of spot-forming cells in response to in vitro recall with RHDV VP60 at days 19, 25 and 40 post-immunization (Figure 7a). The highest frequencies of spot-forming cells in response to VP60 protein were found at day 25 (6 days post-boost), with values of 363.7 ± 122.8 (0.1-mg dose) and 490 ± 144.8 (1-mg dose) spots per million stimulated cells.

In contrast, the immunization of pigs with VLP L-B/N-T did not elicit significant numbers of IFN-γ-producing cells in response to in vitro recall with any of the FMDV-derived antigens (Figure 7a).

Similar results were obtained in lymphoproliferation assays (Figure 7b). Lymphocytes from pigs immunized with either 0.1 or 1 mg of chimeric VLP L-B/N-T elicited a high specific response (SI > 6) against VP60 protein. However, proliferative responses were not detected after in vitro *recall* with peptide T (SI < 2), and a low specific cellular response (SI < 6) was found after in vitro stimulation with peptide B (Figure 7b). Altogether, these results show that the VLP L-B/N-T did not induce a detectable FMDV specific T cell response under the assayed conditions.

#### 3.3.3. Immunization with VLP L-B/N-T Provides Partial Clinical Protection against an FMDV Challenge in Pigs

Protection against FMD was measured by monitoring clinical signs in animals during 10 days after challenge. Rectal temperatures and development of vesicles were recorded daily for quantification of a clinical score (see Methods). Animals were considered protected when lesions were only observed at the inoculation site [38].

As expected, non-immunized control pigs (#7 and #8) showed full FMD signs (vesicles on all four legs) upon challenge, developing secondary lesions on days 4 and 3, respectively (Table 1). These animals developed fever with maximum temperatures of 40.7 °C and 40.0 °C at day 4 post-challenge.

In contrast, while five out of six VLP-immunized pigs showed detectable secondary vesicles, these appeared only by day 7 post-challenge, a relevant delay of 3–4 days when compared with non-immunized controls. Additionally, these five animals showed fewer and smaller vesicles than controls. Remarkably, one animal (pig #2) was considered fully protected, developing only a single vesicle at the virus inoculation point (Table 1). In addition, two out of six chimeric VLP-immunized pigs (#2 and #6) did not develop fever (< 39.5 °C), and two other pigs (#1 and #5) registered fever peaks with temperatures lower than 40 °C. The two remaining immunized pigs (#3 and #4) developed temperature peaks above 40 °C, which in the case of pig #4 occurred at day 6 post-challenge, slightly delayed with respect to control non-immunized pigs (day 4).

**Figure 8:**
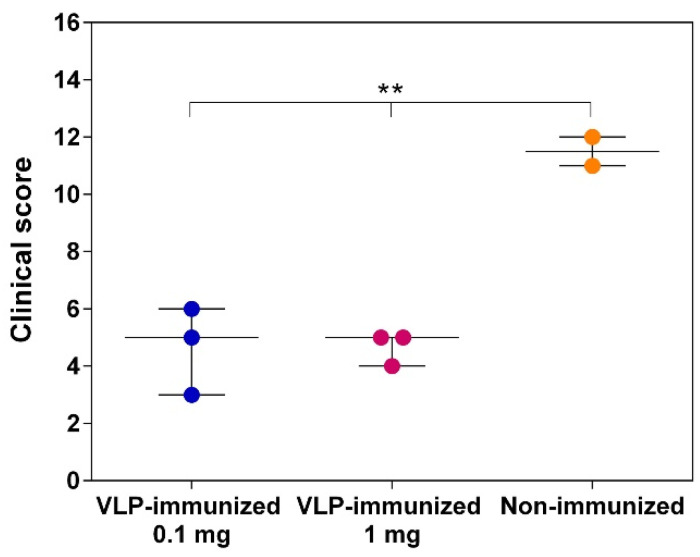
Graph showing the range of the maximum clinical scores (see Materials and Methods) recorded for the individual VLP-immunized pigs, either with 0.1 or 1 mg of chimeric VLP, and non-immunized pigs. Statistically significant differences were found in the median values (line into the box) between both groups (** *p* < 0.01).

The maximum clinical scores (mean + SEM) registered along the post-challenge time course were of 4.66 ± 0.42 for VLP-immunized pigs and 11.50 ± 0.50 for control non-immunized pigs (Figure 8). Overall, the results obtained indicated that immunization with chimeric VLP L-B/N-T significantly reduced FMD severity in pigs after challenge (*p* = 0.0031).

## 4. Discussion

We have developed a system for the generation of VLPs derived from different caliciviruses, such as swine norovirus [42], feline calicivirus [43] and RHDV [25]. Our previous results have shown that RHDV VLPs can be produced to high yields, are highly immunogenic and are suitable to be used as diagnostic reagents or vaccines for the control of RHDV in rabbits [24,41,44]. In addition, we demonstrated that RHDV VLPs are excellent platforms for inducing cellular immune responses against inserted heterologous cytotoxic CD8+ T-cell epitopes [26], as well as potent protective humoral responses against foreign B-cell epitopes in the mouse model [13]. RHDV VLPs are efficiently taken up by dendritic cells [26,45,46], which cross-present foreign T-cell epitopes via the major histocompatibility complex (MHC) class I pathway to initiate cytotoxic T-cell responses [26,47]. Other studies have addressed the potential of RHDV VLPs as a platform for delivery of tumor-associated antigens, demonstrating their ability to elicit protective immunity against tumors [48,49,50].

In this study, we evaluated the ability of engineered VLPs displaying FMDV-derived epitopes to elicit immune response and protection against this relevant livestock disease. To perform this task two well-defined immunogenic epitopes were used. The first one was the immunodominant B-cell site located within the GH loop of FMDV VP1capsid protein from the FMDV O-UKG 11/01 isolate. This epitope has been shown to elicit cross-reactive specific antibodies against a panel of type O FMDV topotypes [51]. The T-cell epitope chosen, located at positions 21–35 of the 3A protein, is highly conserved among different FMDV serotypes and the cellular immune responses elicited against it are heterotypical [33,34]. Previous work had shown that branched structures (dendrimeric peptides) enabling multimeric presentation of the B-cell epitope linked to one copy of the T-cell epitope, elicited efficient induction of neutralizing antibodies, proliferation of T-cells and optimal release of IFNγ, promoting solid protection of pigs against an FMD challenge [33,34,35], while the same two epitopes arranged in a linear peptide configuration were significantly less efficient [36]. Thus, it was of interest to determine whether RHDV VLPs could perform as an effective vaccine vector to deliver both immunogenic FMDV epitopes, inducing protection in pigs.

The five chimeric constructs designed, incorporating one or two foreign epitopes either joined or individually at two different locations within the RHDV capsid protein (involving the insertion of foreign peptide sequences from 19 to 38 aa), were efficiently expressed and readily assembled into VLPs (Figure 1). This result further confirms and expands the reported versatility of RHDV VLPs, which have been shown to be very tolerant not only in accepting foreign sequences at different insertion sites, but also by allowing incorporation of several tandem repeats, spanning at least 62 amino acids [13].

Regarding the induction of humoral responses against the foreign B-cell epitope displayed, the results obtained show that the engineered RHDV VLPs elicited a potent FMDV-specific antibody response, both in mice (Figure 3) and pigs (Figure 6a). Constructs L-B and L-B/N-T, with the B-cell epitope inserted individually at loop L1 exhibited the highest FMDV-specific antibody titers (Figure 3), in agreement with previous reports indicating this surface-exposed insertion site was the best choice for the induction of humoral responses against foreign B-cell epitopes [13], although in this case differences across groups were not statistically significant. These results were in spite of the intrinsic high immunogenicity of the parental RHDV capsid protein, which elicited high anti-VP60 antibody titers (Figure 2), but did not preclude the concurrent induction of potent specific responses directed to the inserted foreign B-cell epitope, as had already been shown with chimeric RHDV VLPs incorporating B-cell epitopes from other viruses, like influenza A and feline calicivirus, at loop L1 [13].

Furthermore, the chimeric VLPs also induced high titers of FMDV neutralizing antibodies in mice (Figure 4). The construct L-B/N-T, which elicited the highest VNT titers in the mouse model, was also shown to induce a robust neutralizing antibody response in pigs (Figure 6b and Table 1) at both doses tested (0.1 mg and 1 mg of chimeric VLP). This FMDV neutralizing immune response was substantially higher than those previously reported for other VLP vector systems like hepatitis B virus, porcine circovirus or bovine parvovirus chimeric VLPs harboring the B-cell epitope within the GH loop of type O FMDV [52,53,54]. Indeed, the induction of neutralizing antibodies anti-FMDV elicited by such chimeric VLPs remains to be demonstrated in natural hosts, as these were only assayed in the mouse model.

Notably, the FMDV VNT titers elicited by VLP L-B/N-T in mice and pigs, were considerably higher than those previously reported for immunization with dendrimeric peptides affording full protection against FMDV challenge [33,34,35,37], as well as those elicited by current chemically inactivated whole-virus vaccines in pigs and cattle [55,56].

In contrast to the powerful induction of specific humoral responses against the FMDV B-cell epitope observed, results obtained with the chimeric VLPs regarding induction of cellular immune responses by the FMDV T-cell epitope displayed were modest. ELISPOT analyses of IFNγ-expressing splenocytes in mice exhibited high frequencies of RHDV VP60-specific IFNγ-secreting cells induced by RHDV VLPs (either parental or chimeric) (Figure 5a). However, the IFNγ responses elicited by chimeric VLPs after in vitro recall with FMDV antigens was detectable but weak (Figure 5b,c). The cellular immune responses elicited by L-B/N-T in the pig model were similar (Figure 7). Strong T-cell responses against parental VP60 protein were revealed both by ELISPOT analyses and lymphoproliferation assays. However, immunization with VLP L-B/N-T in pigs did not elicit significant frequencies of IFN-γ-producing cells in response to in vitro recall with FMDV-derived antigens, and no significant proliferative responses were observed after in vitro recall with the FMDV T-cell epitope. 

In this case, the results obtained with the chimeric VLPs did not match those elicited by the dendrimeric peptides based on the same FMDV immunogenic epitopes, which had been previously shown to be able to induce efficient FMDV-specific T-cell responses [34,35,37]. Several factors might account for the differences observed in the ability of the FMDV T-cell epitope to induce cellular responses, either incorporated to chimeric VLPs or to dendrimeric peptides. T-cell responses require antigen processing to allow presentation of antigenic peptides in association with MHC molecules. Different processing and presentation efficiencies of the T-cell epitope displayed in diverse contexts could explain the results obtained. In the case of the dendrimeric constructs the T-cell epitope was linked to the B-cell branched structure through a cathepsin D cleavage site at its N-terminal end, with a free C-terminus [37], a configuration which might facilitate epitope processing, while in chimeric VLPs the T-cell epitope was incorporated to the VP60 protein flanked by amino acids GS and inserted at different sites within the capsid protein. It has been reported for other VLP systems that flanking sequences greatly influence processing and presentation efficiencies of foreign T-cell epitopes [57]. Therefore, improvement of specific T-cell responses elicited by chimeric VLPs could be attempted by modification of the sequences flanking the inserted epitopes. Another relevant factor might be the presence of immunodominant T-cell epitopes within the RHDV VP60 protein, which could hamper the induction of specific responses by inserted foreign T-cell epitopes. This possibility cannot be ruled out given the demonstrated ability of RHDV VP60 protein to evoke strong IFNγ responses (Figure 5a and Figure 7). We have previously reported the induction of potent specific T-cell responses by chimeric RHDV VLPs displaying chicken ovalbumin (OVA) CD8+ T cell epitope SIINFEKL [26], although it is known that this is a highly immunodominant model epitope, which most likely would not be affected by the presence of VP60 T-cell epitopes. However, certain T-cell epitopes might perform as subdominant when inserted into the VP60 protein. Further research is needed to clarify this possibility, focused on the identification of immunodominant T-cell epitopes within the VP60 protein, which could then be deleted in order to enhance subdominant responses.

Immunization of pigs with L-B/N-T was shown to confer full protection in one out of six immunized pigs, and partial protection to the rest of the animals (Table 1). Partial protection was characterized by a significant delay in the development of lesions compared to non-immunized pigs, as well as by mild clinical signs of the disease. Clinical evaluation of the animals after virus challenge was calculated using a well-established clinical score for FMD infected pigs [35,36,58]. Significant differences on the maximum clinical scores registered were observed between the VLP-immunized pigs and the non-immunized control pigs (Figure 8).

Our results were in agreement with previous reports indicating that, although it is generally accepted that protective immunity to FMDV is mostly due to neutralizing antibodies, a T-cell response is also necessary for effective immunity [59]. 

Chimeric RHDV VLPs elicited strong consistent neutralizing antibody titers in all immunized pigs, which is necessary but not sufficient to induce full protection. The levels of specific T-cell responses required for complete protection were not achieved, thus affording partial protection against FMD challenge. Work is in progress focused on improving the specific T-cell responses induced by chimeric RHDV VLPs displaying FMDV related epitopes.

## Figures and Tables

**Table 1:** Evidence for protection in animals immunized with the chimeric VLP L-B/N-T.

Inoculum	Vaccine Dose (mg)	Pig #	First Day with Vesicles ^1^	Number of Vesicles ^2^	Fever ^3^	SN ^4^ Prime/Boost/Challenge	Protection ^5^
RHDV VLP L-B/N-T	0.1	#1	7	3	39.8 (4)	1.9/2.4/3.4	Partial protection
		#2	7	1	No fever	1.5/2.2/3.6	Full protection
		#3	7	3	40.9 (4)	2.0/2.3/3.6	Partial protection
	1	#4	4	4	40.1 (6)	2.5/2.8/3.7	Partial protection
		#5	7	3	39.9 (5)	2.1/2.4/3.6	Partial protection
		#6	7	3	No fever	1.7/3.6/3.4	Partial protection
Non-immunized	-	#7	4	4	40.7 (4)	0/0/3.0	Non-protected
		#8	3	4	40.0 (4)	0/0/2.9	Non-protected

^1^ First day post-challenge with detectable secondary lesions (vesicles); ^2^ total number of vesicles (vesicular lesions at inoculation site, feet, tongue, mouth and nose); ^3^ temperature (°C) and day post-challenge when maximum temperature was registered. No fever (temperature ≤39.5°); ^4^ serum neutralizing antibody responses reported as log10 TCID50 at days 19, 67 (48 days post-boost) and 77 (10 days post-challenge). Log10 SN values ≤0.6 are indicated as 0; ^5^ animals were considered protected when lesions were only observed at the infection site [38]; partially protected when animals showed mild and delayed signs of disease.

## Data Availability

All the data that support the findings of this study are included within the article.
